# Cupid, a cell permeable peptide derived from amoeba, capable of delivering GFP into a diverse range of species

**DOI:** 10.1038/s41598-020-70532-x

**Published:** 2020-08-13

**Authors:** Daniel Fenton, Dylan Phillips, Anne Maddison, Christopher H. George, Jonathan Ryves, Huw D. Jones

**Affiliations:** 1 Institute of Biological, Environmental and Rural Sciences (IBERS), Aberystwyth University, Penglais, Aberystwyth, Ceredigion, SY23 3DA Wales, UK; 2 Institute of Life Sciences, Swansea University Medical School, Singleton Park Campus, Swansea, SA2 8PP Wales, UK; 3 Cupid Peptides, Cardiff Medicentre, Heath Park, Cardiff, CF14 4UJ Wales, UK

**Keywords:** Biochemistry, Biotechnology, Cell biology, Drug discovery, Molecular biology, Molecular medicine

## Abstract

Cell permeating peptides (CPPs) are attracting great interest for use as molecular delivery vehicles for the transport of biologically active cargo across the cell membrane. The sequence of a novel CPP sequence, termed ‘Cupid’, was identified from the genome of *Dictyostelium discoideum*. A Cupid-Green Fluorescent Protein (Cupid-GFP) fusion protein was tested on mammalian, whole plant cells, plant leaf protoplast and fungal cell cultures and observed using confocal microscopy. GFP fluorescence builds up within the cell cytosol in 60 min, demonstrating Cupid-GFP has permeated them and folded correctly into its fluorescent form. Our combined data suggest Cupid can act as a molecular vehicle capable of delivering proteins, such as GFP, into the cytosol of a variety of cells.

## Introduction

The cell membrane or plasma membrane surrounding the cytoplasm of living cells is selectively permeable to ions and organic molecules, and acts as a barrier to regulate what enters and exits the cell. It is made mostly from a bilayer of phospholipid molecules with a variety of protein molecules embedded within it and, depending on the type of organism, it may be further bounded externally by a cell wall^1,2^.

Cell-permeable or cell-penetrating peptides (CPPs) (also known as a protein transduction domain or membrane translocation sequence) are increasingly being used to overcome the impermeability of the plasma membrane. Their ability to traverse cell membranes and access the cell interior allows them to be exploited to internalize molecules (generically termed ‘cargo’) such as proteins, oligonucleotides, peptides, nucleic acids, and other pharmacologically active compounds into a range of living systems. Thus, CPPs provide a promising strategy for cellular delivery of otherwise membrane-impermeable molecules for research and therapeutic purposes^3–10^. Understanding how CPPs interact with membranes and achieve access to the cell interior is an on-going area of research.

CPPs may be classified based upon their binding properties to lipids linked to their peptide sequences: primary amphipathic; secondary amphipathic; and non-amphipathic^11–13^. They can also be classified according to the physical mechanism of cellular uptake, for example whether it is endocytosis-mediated or direct permeation of the membrane.

For endocytosis-mediated delivery, CPPs are thought to first interact with the outer surface of the cell membrane via molecular charge or binding to surface proteoglycans, glycosaminoglycans or other receptor before being internalized by the endocytotic machinery. Macropinocytosis, clathrin-coated pits and caveolae/lipid-raft-mediated endocytosis mechanisms have been implicated through their differing sensitivity to drug applications^14–19^. These processes involve energetic membrane folding and employ internalization proteins that require cellular ATP to function. Direct permeation of the membrane involves a mechanism that is independent of classic endocytosis, requiring no cell surface receptor or cell-derived ATP energy to do so. It is thought to occur without the requirement of the cell internalization machinery and has been proposed to involve sequential steps of binding (localization) then insertion into the bilayer followed by translocation across the membrane by ‘flipping’ or inverted lipid micelle formation. Interactions between CPPs and the phosphate groups on both sides of the lipid bilayer, the strength of the transbilayer potential and the formation of a transient pore have been suggested to play roles in these steps^20–25^. However this process, especially with respect to permeation of CPPs with large cargoes, remains to be fully elucidated at the molecular level. Additionally, the mode of cell permeation may be influenced by other factors such as the CPP concentration and the size and type of the cargo attached to the CPP. It has been suggested that small cargoes are capable of utilizing a non-endocytic pathway, whereas larger ones tend to use the endocytic delivery^26–28^.

Finally, the route of permeation will directly affect the bioavailability of cargoes within the target cell since endosomal trapping can lead to lysosomal degradation hence limiting cargo access to other intracellular targets^29–31^ unless further protein engineering can facilitate subsequent endosomal escape^32^.

It is well accepted that fluorescent proteins such as GFP, whether denatured or folded into their functional fluorescent form, do not permeate cell membranes unaided^33^. This paper introduces a novel cell permeating peptide sequence identified in the genome of the free-living amoeba, *Dictyostelium discoideum*, termed ‘Cupid’, (*C*ell*u*lar *P*ermeability factor *i*n *Dictyostelium*). Using confocal fluorescence microscopy we report here on the capability of Cupid to deliver the 29 kD protein, GFP, into the cytosol of cultured eukaryotic, whole plant cells, plant leaf protoplasts and fungal cells.

## Materials and methods

### Identification of Cupid peptide sequence

The *Dictyostelium discoideum* genome^34^ was searched for possible penetratin peptide sequence (RQIKIWFQNRRAKWKK)^3^. After failing to find any identical matches, a degenerative BLAST search identified a putative CPP sequence in the hypothetical protein DDB_G0284293 (NCBI Reference Sequence: XP_638704.1). The 16-mer peptide (RRVQIWFQNKRAKVKR), which we termed CUPID, was tested for cell penetrating activity.

### Generating Cupid peptides

**Figure 1:**
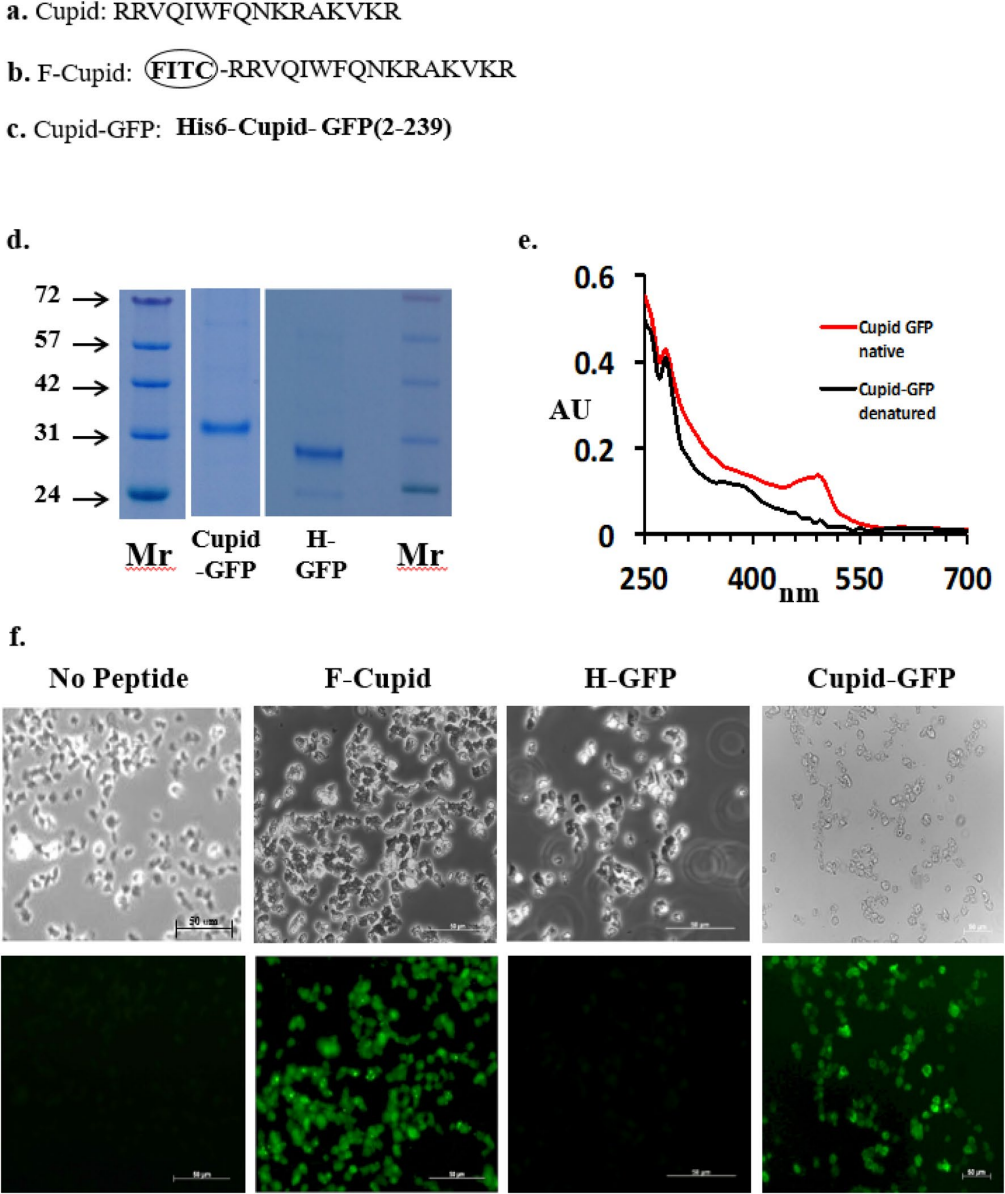
(**a**) Cupid sequence. (**b**) Fluorescein labelled Cupid (F-Cupid). (**c**) Cupid-GFP. (**d**) Cupid-GFP (4 µg) and H-GFP (4 µg) after SDS-PAGE gel stained with Instant blue. Molecular weight markers (Mr) shown in kD. (**e**) Absorbance Spectrum wavelength in nanometres of native (fluorescent) GFP (red) and Cupid-GFP after resolubilisation (denatured /non-fluorescent) (black). (**f**) *Dictyostelium* attached to chambered slides were treated with: no peptide, 10 µM F-Cupid**,** 10 µM H-GFP or 10 µM Cupid-GFP for 1 h. Following washing steps, cells were fixed and observed with phase (Upper Panel) or green fluorescent (Lower Panel) filters.

N terminal FITC-Cupid (RRVQIWFQNKRAKVKR) was synthesized by Severn Biotech Ltd, UK and diluted in buffer (10 mM Tris). For Cupid-GFP, a pBR322-based plasmid was constructed to encode GFP from *Aequorea victoria* (Jellyfish) (UniProtKB/Swiss-Prot: P42212.1). The GFP cassette incorporated sequence encoding amino acids 2–239 of GFP (ie missing the initial methionine) a Hisx6 N-terminal tag and the Cupid sequence (RRVQIWFQNKRAKVKR). This plasmid was used to transform the *E. coli* strain BL21(DE3) which was grown overnight to mid-log phase at 37 °C in LB broth supplemented with Ampicillin (100 µg/mL). Cupid-GFP peptide production was induced with 0.5 mM IPTG for 3 h and the bacteria harvested by centrifugation. Bugbuster Reagent (Novagen, UK) was used to lyse the pelleted cells before passing the lysate through Ni–NTA His Bind Resin (Novagen, UK). Bound peptide was washed and eluted under either native or denaturing conditions (according to manufacturers recommendations) then dialyzed overnight. Peptides were dried and stored at − 20 °C until use, when they were resuspended at a stock concentration of 100 µM in 10 mM Tris buffer (at the pH of the buffer system of the cells undergoing experimentation) or alternatively distilled sterile water. Peptide purity was assessed by SDS-PAGE analysis and protein concentration was determined using a Pierce BCA Protein Assay Kit (Life Technologies, UK). The fluorescent spectrum of native or denatured Cupid-GFP peptide in 10 mM Tris buffer solution pH 8 was assessed using a NanoDrop spectrophotometer (Thermo Scientific, Rockford, USA). For control experiments, His-tagged-GFP (H-GFP) lacking the Cupid CPP, was constructed and purified, stored and resuspended in a similar manner. H-GFP migrated as a single band at the expected mass of 29.2 kD on SDS gels (Fig. 1 and Supplemental Fig. 4).

### Spectrophotometric absorbance of native and denatured Cupid-GFP

Native and denatured Cupid-GFP were incubated for 24 h at 37 °C and room temperature in the following cell-culture media: DMEM, Claycomb, phosphate-buffered saline and W5. After 24 h the fluorescence spectrum of native or denatured Cupid-GFP peptide in 10 mM Tris buffer solution pH 8 was assessed using a NanoDrop spectrophotometer (Thermo Scientific, Rockford, USA). For each treatment, the machine was blanked, as appropriate, against the relevant media used.

### Amoebae cell preparation and imaging

*Dictyostelium discoideum* AX2 strain cells were grown on *Klebsiella aerogenes* plates^35^. For experimentation, cells at the growth zone were collected and washed 5 times with 10 mM potassium phosphate buffer, pH 7.2 and seeded in slide chambers at a density of 10^5^ cells/mL. Cells were incubated with FITC-labelled Cupid, Cupid-GFP or H-GFP at the doses indicated for 60 min, washed 3 times in buffer and fixed in situ using Fluorsave (Calbiochem, UK) under a coverslip. Cell microscopy was performed on an upright fluorescence microscope (Olympus BX61) with the green fluorescent filter set, coupled with a digital camera.

### Mouse cell cultures and imaging

Murine HL-1 cardiomyocytes were cultured on a gelatin (0.02% w/v)/fibronectin (10 µg/ml) (GF) matrix in Claycomb medium supplemented with 10% fetal bovine serum (FBS), norepinephrine (0.1 mM), L-glutamine (2 mM) and penicillin/streptomycin antibiotics (100 units/ml, 100 µg/ml, respectively). Cells were maintained in a humidified 37 °C incubator in 5.0% CO_2_ atmosphere. For imaging work HL-1 cells were seeded on 30 mm GF-coated glass-bottomed dishes (Mattek) at 80% confluency and cultured to confluence (96 h). Prior to the start of experiments, media containing peptide/recombinant protein was added to the cells.

The confocal microscopy on live cultured mouse cells was performed using a Leica SP5 TCS system equipped with a HCX PL APO CS 63× objective (numerical aperture 1.4). For excitation of GFP signal a 488 nm laser line of an Argon laser set at 30% maximum power, further attenuated to 6% power using an acousto-optical tuning filter (AOTF) was used. Scanning at 8000 Hz GFP fluorescence emission between 510–550 nm was collected over a 512 × 512 pixel array 145 × 145 µm^2^ with images produced by averaging 4 times per line and 4 frames. Z-stacks were collected over approximately 32 µm depth with each Z section being separated by 1.09 µm. Data were collected using photomultiplier tubes (PMT) set at 8-bit resolution (i.e. pixel intensity ranged from 0–255). Leica LAS software was used to process the raw image files. Bleed through between the channels was avoided using sequential scanning with photomultiplier and laser settings remaining constant within each experiment. Optimization of PMT offset and gain was performed to the same extent within each experiment making the image panels comparable.

### Plant cell preparation and imaging

A 1 × 1 cm square was cut out from an onion bulb (*Allium cepa* L.) and scales carefully separated into individual layers with forceps. The epidermal cells comprising a thin film on the inside of each layer, was peeled off and flattened onto glass slides. To this layer was added 20 µL of either peptides (5 µM) or water, drop by drop, and then a coverslip was firmly pressed onto the film, flattening it as much as possible, before inverting the glass slide and placing it under the microscope. Because the film of cells is very thin, approximately 1 or 2 cells thick, dropping the media on top of it and then adding a cover slip was enough to immerse the whole epidermal layer in fluid.

Protoplasts were isolated from the leaf tissue of 25-day-old *Brachypodium distachyon* plants by the enzymatic methodology of Jung et al.^36,37^. The final preparation of purified protoplasts was resuspended in 5 mL of filter-sterilized W5 solution (0.037% (w/v) KCl, 0.9% (w/v) NaCl, 1.84% (w/v) CaCl2, 2 mM MES-KOH pH 5.7).

Confocal live plant and fungal cell microscopy was performed on a Leica TCS SP5 II confocal system using a Leica DMI6000 inverted microscope stand. For this study we used a DPSS 561 nm laser, an Argon laser, PL APO 40x/1.25—0.7 and HCX PL APO 63x/1.40—0.6 (oil) objectives, GFP (green) fluorescent filters, and LAS AF version 2.7.3.9723 software to control the microscope, scanning, laser module, tools and the image acquisition and processing.

### Fungal cell preparation and imaging

The wild-type strain of zygomycetous fungus *Phycomyces blakesleeanus* (Burgeff) NRRL 1555(–) was used in this study on solid agar medium supplemented with yeast extract (0.1%) and bacto-casitone (0.1%), with agar at 1.5% (w/v) in 10 cm petri dishes^38^. A 0.5 × 0.5 cm block of agar from a plate infused with a growing *P. blakesleeanus* was excised and placed in contact at the side of a sheet of agar 2 cm × 2 cm × 0.5 cm. After 4 days the fungal mycelia network had invaded this sheet, and a 0.5 × 0.5 × 0.5 cm cube was selected, excised and placed on a microscope slide. 70 µL of either water or peptide (5 µM, non-fluorescent) was added drop by drop, allowing it to soak into the agar. After waiting 30 min, a coverslip was firmly pressed onto the agar. After 90 min the slide was inverted and imaged using the system above (see Plant Cell work). Three time lapse videos were created to show the Cupid-GFP interacting with the cytoplasmic flow within the mycelia network (4 min time lapse, 8 s imaging interval) (see Supplemental Videos 1, 2 and 3).

### Image data analysis

Raw images were assessed for increase of GFP fluorescence using Image J software (NIH Image, Version 10.2) and average values + /− SEM were compared with the untreated controls to produce time-course graphs of Cupid-GFP uptake.

## Results

The 16 amino acid Cupid sequence (Fig. 1a) was synthesized with a fluorescein molecule attached at the N terminal (F-Cupid, Fig. 1b). When added to *Dictyostelium* amoeba cells in buffer at 10 µM, F-Cupid caused the cells to become fluorescent within 60 min while fluorescein-labelled BSA did not (data not shown), indicating cell-penetrating capability (Fig. 1f).

To investigate the nature of this capability, an expression plasmid was constructed to link the carboxy-terminus of the Cupid peptide to the *Aequorea victorea* Green Fluorescent Protein (GFP) sequence, and the fusion protein was purified using an N terminal polyhistidine motif (see “Materials and methods” section, Fig. 1c). This Cupid-GFP protein migrated as a single band at the expected mass of 32.3 kD on SDS gels (Fig. 1d and Supplemental Fig. 4) and under the conditions of purification was non-fluorescent, indicating a denatured state (Fig. 1e). However when we tested the native, fluorescent Cupid-GFP, it aggregated in the culture medium and the fluorescent peptide did not penetrate the cells (Supplemental Fig. 2). Therefore, all subsequent experiments were conducted with denatured, non-fluorescent peptides. Furthermore, when the dried denatured peptides were solubilised (in either water, phosphate-buffered saline, DMEM, Claycomb or W5 media) they remained non-fluorescent for at least 24 h at room temperature or at 37 °C (Supplemental Fig. 3). When amoeba were treated with Cupid-GFP (10 µM, 1 h), they became fluorescent (Fig. 1f) demonstrating the GFP cargo refolds to its fluorescent form under the physiological conditions within the cell. Denatured H-GFP failed to demonstrate the same permeability/fluorescence profile.

These initial experiments in amoeba used the fixative reagent Fluorsave prior to imaging which could have altered the subcellular distribution of Cupid-GFP as has previously been shown for other CPPs^39^. We therefore turned to confocal fluorescence microscopy of living, unfixed cells to examine the extent of cupid GFP permeability and localization.

**Figure 2:**
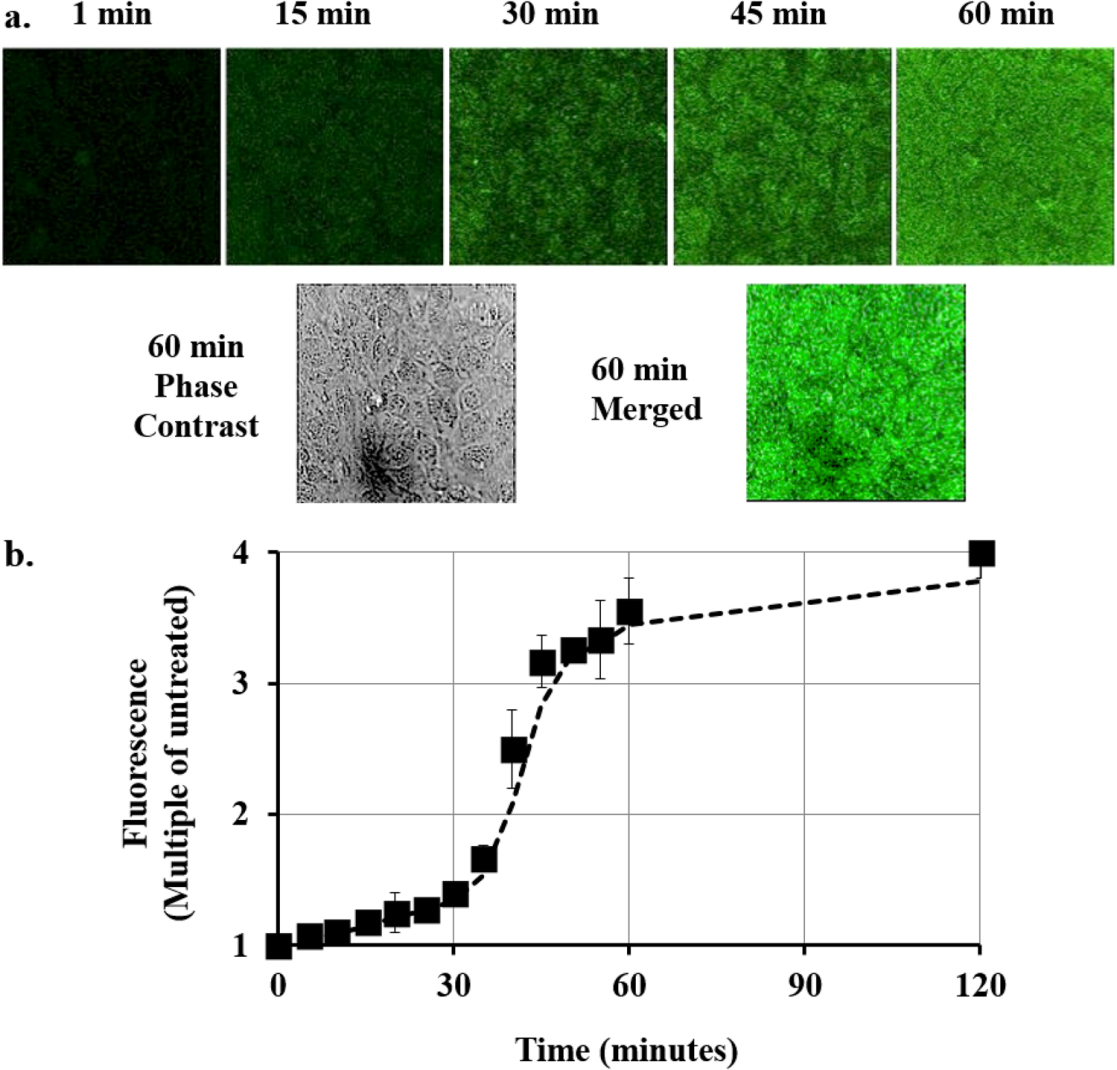
(**a**) Total Z-Stack confocal imaging of confluent mouse cardiomyocytes. Top panel: Culture treated with 5 µM Cupid-GFP and imaged for Total green fluorescence at times indicated. Lower Panel: Phase contrast image and merged image at the 60 min time point. (**b**) Increase in GFP fluorescence in mouse cardiomyocytes after addition of 5 µM Cupid-GFP. The Z Stack green fluorescence at each time point was plotted as a multiple of Z Stack green fluorescence of untreated cells. Error bars + /− SEM N = 4.

GFP fluorescence was monitored in confluent cultures of mouse cardiomyocytes using Cupid-GFP at a dose of 5 µM and imaging total GFP fluorescence over time (Fig. 2). As the added Cupid-GFP was initially non-fluorescent, it was possible to image the cells at 5 min intervals without disturbing the plate by washing. The data were used to plot the overall increase in fluorescence with time, relative to the background (Fig. 2b). The data indicated fluorescence accumulation progressed through 3 phases: an initial slow phase up to 30 min followed by a rapid phase to 60 min and a final slow phase to saturation at 2 h. Confocal z-slices through the cells at the 60 min time point found GFP to be evenly distributed throughout the internal cell architecture and not confined to discrete vesicles (Supplemental Fig. 1).

**Figure 3:**
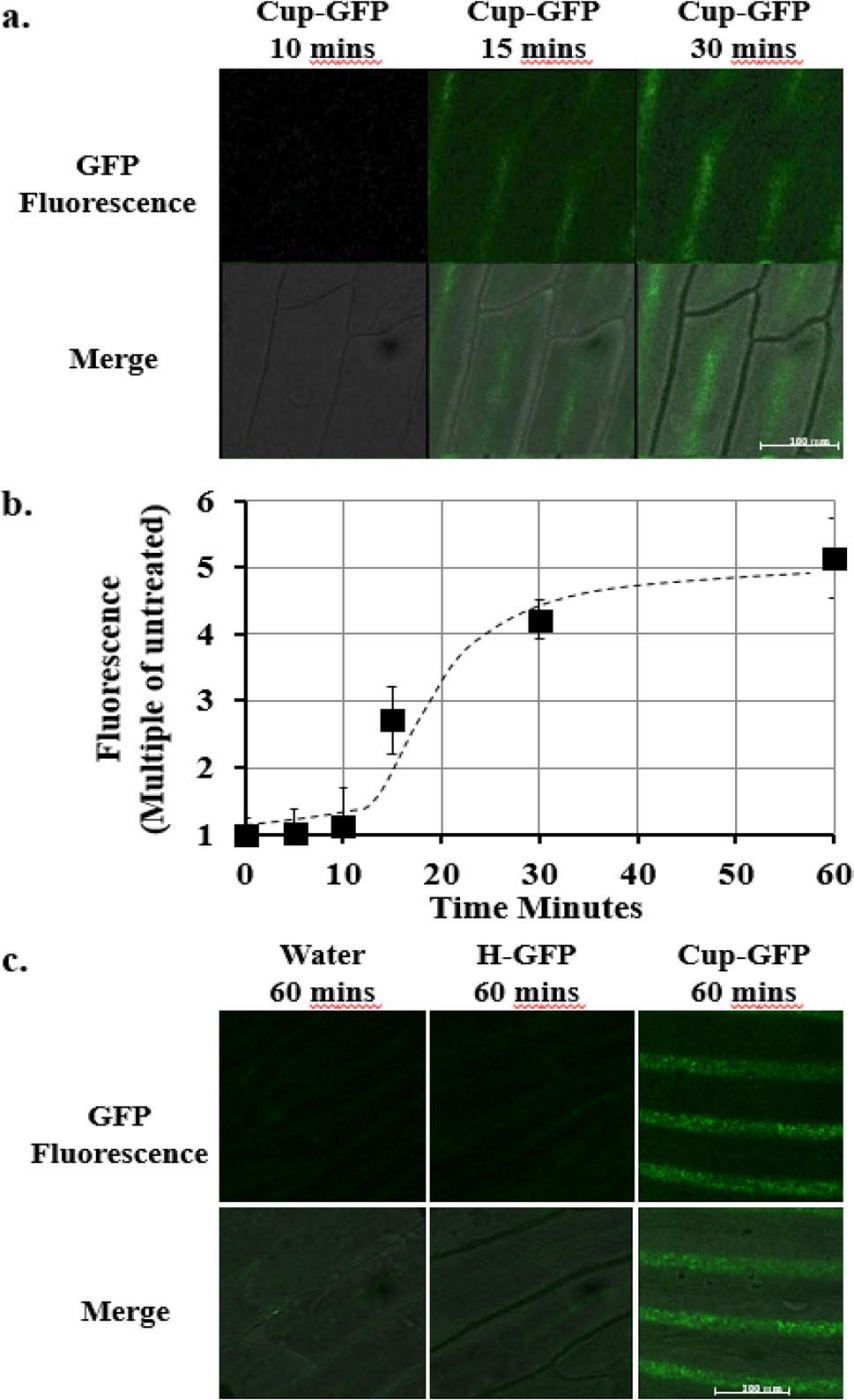
(**a**) Total Z-Slice confocal imaging of *Onion* epidermal cells at 10, 15, 30 min after addition of 5 µM Cupid-GFP. Living plant cells were observed with green fluorescent (Upper Row) filter set and this was merged with phase images (Lower Row). (**b**) Increase in GFP fluorescence in *Onion* epidermal cells after addition of 5 µM Cupid-GFP. The Total Z Stack green fluorescence at each time point was plotted as a multiple of green fluorescence of untreated cells. Error bars +/− SEM N = 4. (**c**) Total Z-Slice confocal imaging of in *Onion* epidermal cells at 60 min after addition of Water, 5 µM H-GFP or 5 µM Cupid-GFP. Living plant cells were observed with a green fluorescent (Upper Row) filter set and this was merged with phase images (Lower Row).

In the next set of experiments, the penetration of Cupid-GFP into plant and fungal cells was assayed. For intact plant cells, onion epidermis was prepared on a glass slide and treated with peptide before a cover slip was applied. After a lag phase, the onion epidermal cells developed strong fluorescence over background which increased with time (Fig. 3a) to a maximum at 60 min (approx. sixfold, Fig. 3b). Onion cells treated with the H-GFP protein (His tagged GFP, identical to Cupid-GFP but lacking the Cupid motif) failed to develop fluorescence above the level seen in water treated cells (Fig. 3c).

**Figure 4:**
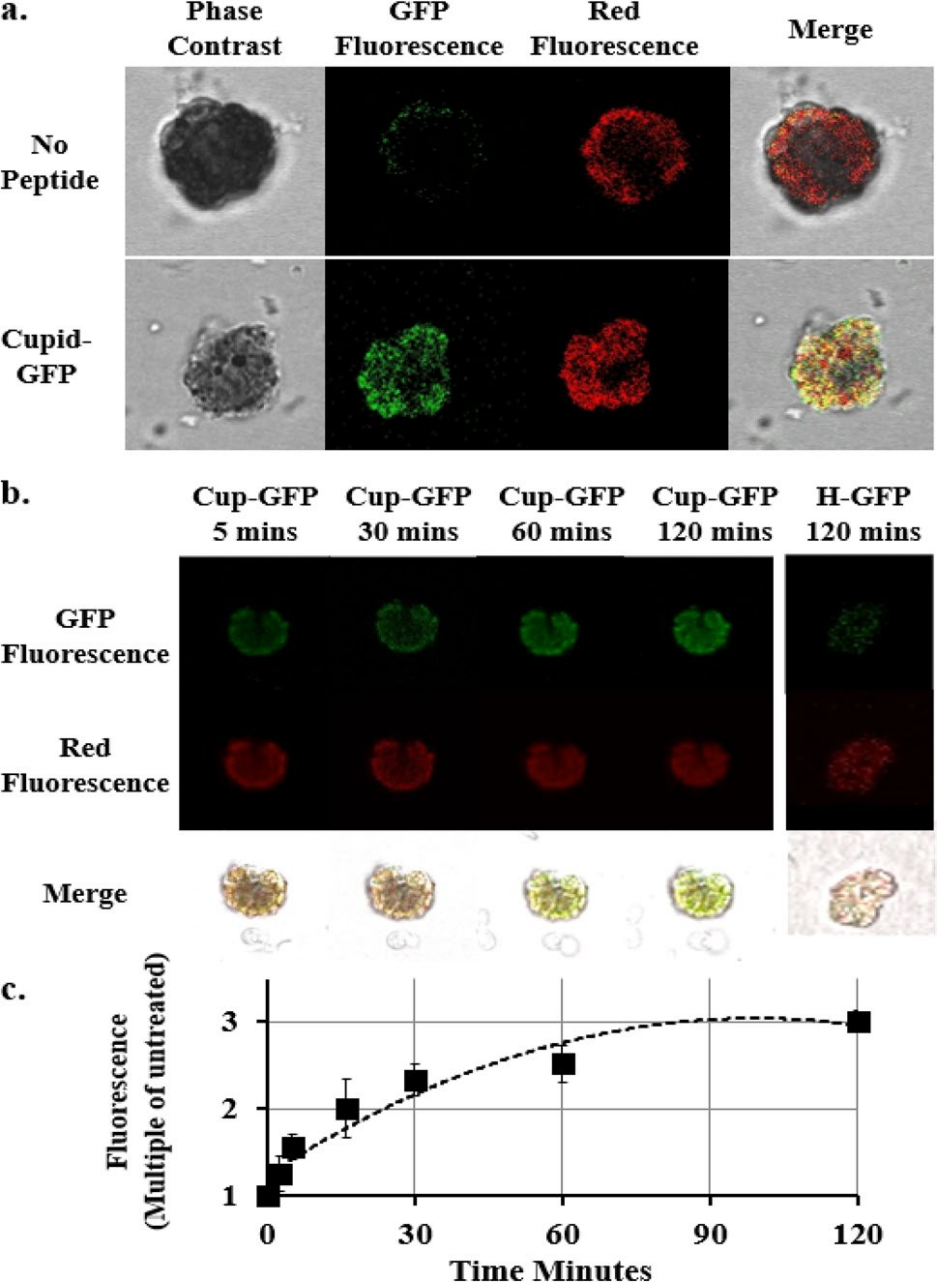
(**a**) Mid-cell Z-Slice confocal imaging of *Brachypodium* leaf protoplasts at 1 h either in the absence (Upper Panel) or presence (Lower Panel) of 5 µM Cupid-GFP. Living protoplasts were observed with phase, green fluorescent and red fluorescent filters. (**b**) Mid-cell Z-Slice confocal imaging of *Brachypodium* leaf protoplasts at 5, 30, 60 and 120 min after addition of 5 µM Cupid-GFP or Control H-GFP at 120 min (right panel). Living protoplasts were observed with a green fluorescent (Upper Row) or red fluorescence (Middle Row) filter set. Lower Row: merge of green, red and phase images. (**c**) Increase in GFP Fluorescence in *Brachypodium* leaf protoplasts after addition of 5 µM Cupid-GFP. The Total Z Stack green fluorescence at each time point was plotted as a multiple of green fluorescence of untreated cells. Error bars +/− SEM N = 4.

*Brachypodium* was selected as the test system for plant protoplasts, where the outer cellulose layer of plant cells are enzymatically removed and the resultant protoplasts are recovered through density sedimentation and sieving, since these have been established as a model system for studying and manipulating plant physiology. Here protoplasts were prepared from *Brachypodium* leaves and treated with Cupid-GFP at a dose of 5 µM. Protoplasts treated with Cupid-GFP quickly developed GFP fluorescence over background and compared to the auto-fluorescence captured in the red channel observed in all protoplasts (Fig. 4a). This GFP fluorescence increased with time (Fig. 4b), whereas protoplasts treated with the H-GFP protein failed to develop any fluorescence above control. The total fluorescence increase was calculated relative to untreated control and data compiled into a graph (Fig. 4c), showing Cupid-GFP caused a rapid increase in fluorescence tending toward a plateau after 2 h.

**Figure 5:**
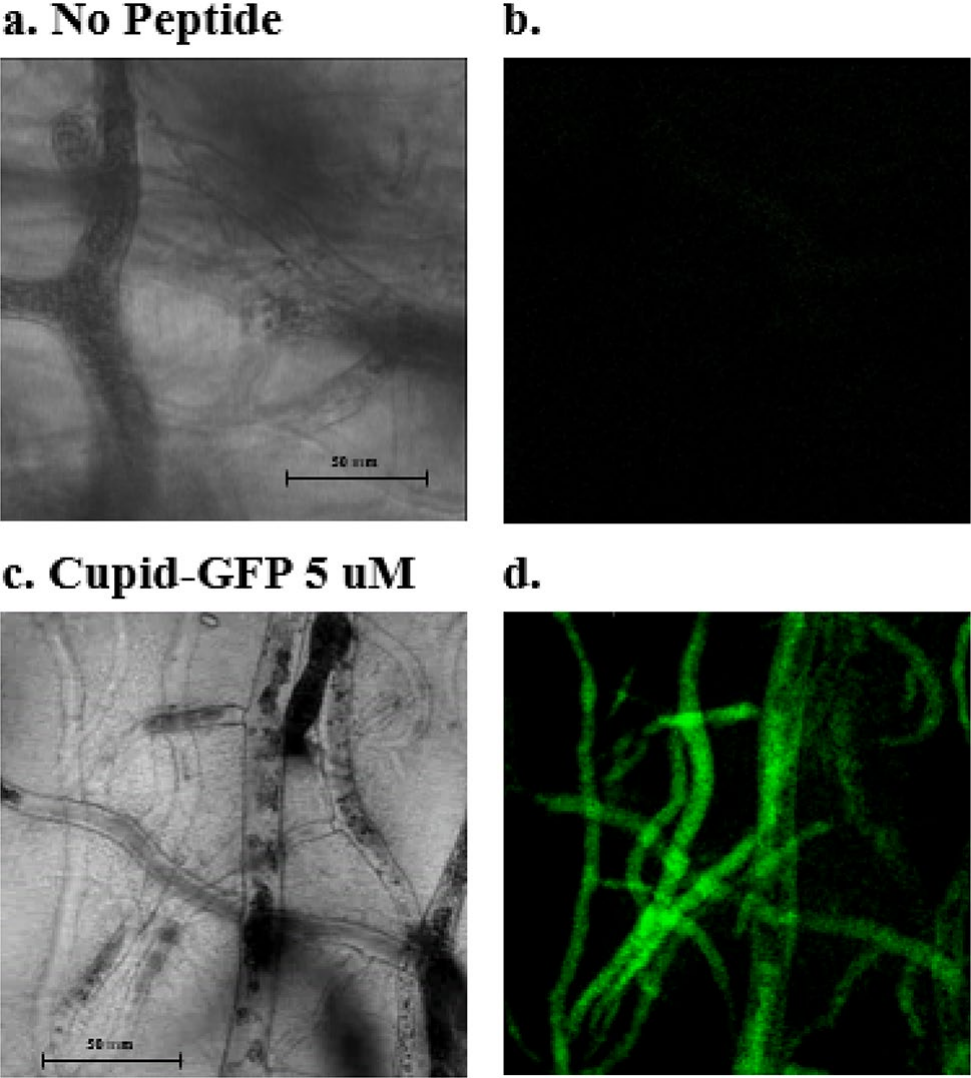
*Phymocyces blakesleeanus* mycelia network within a 5 mm deep agar block was treated with no peptide (**a, b**) or 5 µM Cupid-GFP (**c, d**) for 90 min. A Z-plane was observed with phase (**a, c**) or green fluorescent (**b, d**) filters. See Supplemental Video.

To observe the effects of Cupid-GFP in fungi, the model organism P. *Blakesleeanus* was chosen. For experiments to monitor cell penetration, a slab of agar in which P. *Blakesleeanus* was growing was soaked in non-fluorescent Cupid-GFP (5 µM, 70 µL) and left for 90 min. The slab was then mounted for microscopy and the mycelia network was observed to be strongly fluorescent (Fig. 5). A 1 min 42 s time-lapse movie (8 s imaging interval) with both phase and fluorescent filters showed GFP fluorescence to be moving rapidly through the mycelia network in the living fungus (Supplemental Video 1, 2 and 3).

## Discussion

Although natural cell permeable peptides (CPPs) are present in diverse species, the potential for sequences to act as CPPs is unpredictable^40^ and none have yet been shown in *Dictyostelium* amoeba. This suggests either the amoebae have no requirement for a protein transduction mechanism via a CPP, or it might achieve this end with a cryptic CPP sequence. The present study reports the identification and characterization of a novel CPP derived from a homeobox transcription factor in the slime mould *Dictyostelium discoideum*, termed Cupid (*C*ell*u*lar *P*ermeability factor *i*n *Dictyostelium*) to denote its origin.

The Fluorescein-labelled 16-mer Cupid peptide was found to be efficiently taken up by amoeba cells suggesting that the Cupid sequence is a CPP. It is known that the attachment of dye can influence cell penetration characteristics^41^ so to test whether Cupid can translocate protein cargos into cells, Cupid was fused to GFP. When correctly folded, GFP forms a nearly perfect cylinder and three central amino acids within this barrel structure are spontaneously modified into a chromaphore responsible for fluorescence of the protein. Unfolding this GFP barrel structure repositions the amino acids surrounding the chromophore region, and abolishes fluorescence in a reversible manner^42,43^.

The peptides used in this study were purified under denaturing conditions and thus were not initially fluorescent. Crucially, this approach meant the rise in GFP fluorescence within cells could be studied with live confocal imaging without the requirement to aspirate the cell medium or wash the cells. It also avoided imaging artefacts due to cell fixation, that been observed in other CPP studies and can lead to serious problems in interpreting data^39,44^.

Live confocal imaging reveals that cultured mouse cells, whole plant cells, plant leaf protoplast and fungal cells rapidly exhibit GFP fluorescence when Cupid-GFP is present in the media. As the Cupid-GFP is initially non-fluorescent, we were able to assess total fluorescence in situ without washing steps. In mouse cells, the initial appearance of GFP fluorescence develops slowly over the first 30 min after which it increases rapidly to 45 min and then continuing to increase more slowly to reach a plateau after 60 min. This non-linear increase in GFP fluorescence is likely to be the net effect of different processes occurring in the cell including membrane association, CPP permeation and protein refolding. These processes are likely to operate with different kinetics in different cell types and require further work to elucidate fully. Although no gross membrane lysis was observed in any cell types tested, possible temporary or localised membrane disruption cannot be excluded.

Although accurately relating GFP fluorescence to concentration within cells is difficult, using the paper by Niswender et al.^45^ as a guide our raw data indicates Cupid-GFP instigates a four-fold increase on basal fluorescence at the later time points, suggesting that from an external concentration of 5 µM, the internal concentration of Cupid-GFP has reached as high as 3 µM in mouse cardiomyocytes. This is approximately tenfold higher than calculated for a range of other CPPs linked to the SNAP-Tag system (Mr approx. 20–25 kD), labelled with Rhodamine dye and investigated with Fluorescence Correlation Spectroscopy in mammalian cells^46^.

Previous studies have fused GFP to a variety of CPPs and tested whether GFP can be taken into mammalian cells and, if so, explored the method of entry^31,39,47–50^. Using VP22 (Human Herpes virus protein), TAT (HIV-1 transcriptional activator Tat protein), Polyarginine/Polylysine or Penetratin, these studies reported CPPs first adhered to the cell membrane before being endocytosed within small vesicles. Cellular uptake of GFP modified to contain a cationic “patch” but with no linked CPP has also been detected^28,33^. The authors conclude this was dependent on cell-surface glycosaminoglycans, similar to that observed with cationic CPP peptides such as polyarginine^28,33^. In contrast to these earlier reports, using the ability of the confocal microscope to look through cell sections, we saw no evidence of punctate GFP fluorescence that would be characteristic of endocytotic vesicles in the mouse, plant or fungal cells.

Studies performed with CPPs in plants and fungi are less numerous in the literature. Our initial studies were conducted on onion epidermal layer followed by a more detailed study with protoplasts. The Cupid-GFP readily penetrated all cells in the onion layer, indicating that the peptide was able to pass through the cellulose-hemicellulose network, embedded in the pectin matrix of the plant cell wall. The ability of a CPP-linked GFP to penetrate plant cells broadly agrees with a previous study on onion cells^51^. However, whereas fluorescence of TAT-GFP and polyarginine-GFP in this earlier study appear more concentrated at the cell periphery, which might indicate endocytotic entrapment, Cupid-GFP fluorescence appears to be more uniformly spread throughout the onion cell. The relative absence of endogenous fluorescence in onion epidermal cells compared to mammalian and protoplasts derived from plant leaves is likely reflected in the greater fold-increase in fluorescence from baseline with Cupid-GFP treatment compared with these other systems.

Where other researchers have genetically treated protoplasts to express GFP alone (e.g.^52,53^) the nascent GFP fluorescence is seen encircling chlorophyll-dense structures but not inside them. In contrast using confocal imaging, we see a strong localization of Cupid-GFP and chloroplast auto-fluorescence when GFP/Red fluorescence data is merged (Fig. 4). This indicates that Cupid-GFP has not only reached the plant cytosol but has also penetrated structures that hold the chloroplasts. The cellular machinery to refold proteins, and thus Cupid-GFP, may be concentrated within chlorophyll containing structures, which might account for its close association with such. Also seen within protoplasts but not in mammalian cells are water vacuoles, inner spaces observed as clear patches when using red fluorescence confocal microscopy (e.g. Fig. 4). GFP is also absent in these same areas which could be due to these vacuoles lacking the cell machinery to refold Cupid-GFP. It might also provide evidence that, having entered cells, and once it has refolded, Cupid-GFP can no longer penetrate through additional membranes. Alternatively, there might be other factors at play in the vacuoles to suppress GFP fluorescence, such as pH.

Previous work has found CPPs to be inefficient in entering plant protoplasts and microspores compared with mammalian cells^54,55^. These studies used a wide range of CPPs (polyarginine, pVEC (derived from a murine vascular endothelium cadherin), penetratin, transportan-10 (TP10) and model amphipathic peptide (MAP) either labelled with small molecule dyes or fluorescent proteins (e.g. mCherry). Conjugates that entered cells in these studies exhibited fluorescence patterns consistent with attachment to outer membranes leading to the conclusion that CPP–macromolecular cargo complexes and conjugates were translocated via macropinocytosis (Reviewed by^56^).

In contrast to these studies, we find that Cupid-GFP is taken up by protoplasts and rapidly folded to fluorescent form throughout the cytosol. Whilst the quantity of Cupid-GFP that the protoplasts accumulate is similar to that seen in mammalian cells when applied at 5 µM (threefold vs. fourfold as judged by respective auto-fluorescence), this fluorescence emerges within protoplasts without the lag phase apparent in mammalian or whole plant cell systems. This could be a consequence of the protoplast purification method (e.g. enzymatic stripping of the cell membrane might clear the membrane of interfering proteins and lead to a more rapid entry by shortening an initial Cupid-membrane association time) or it might be that the Cupid-GFP folding machinery in plant protoplasts is more efficient than in the mammalian and whole plant cells. Since protoplasts of some species may be regenerated into whole plants, the data presented here indicate an opportunity to use Cupid within the window of protoplast viability to quickly introduce novel proteins to influence the plant biochemical profile and complement genetic manipulation of plant systems.

Investigation in fungal cells has mainly been concerned with probing the anti-fungal properties of CPPs and comparing them with a range of pore-forming antimicrobial peptides^57–60^. More recently, translocation of a wide panel of Fluorescein dye-labelled CPPs has been investigated in *Candida albicans*^61,62^. These results suggested that penetration into fungal cells involved both endocytosis and membrane permeabilisation. When CPPs have been fused to larger proteins such as GFP, however, the viability of *Candida* cells was not significantly affected^63^ suggesting that CPPs might perform as vehicles to deliver cargoes with the broader intention of investigating these organisms.

We report here how Cupid-GFP, applied externally onto agar in which *P. Blakesleeanus* is growing, quickly penetrates through the chitin coating of the fungus and refolds within the shared mycelia cytoplasm. The refolded GFP observed with time-lapse fluorescence microscopy reveals dynamic anterograde and retrograde movement of nuclei, mitochondria, vacuoles and vesicles through hyphae trunks of *P.Blakesleeanus*. This bulk cytoplasmic flow has previously been observed in fungi using dyes or genetic mutants synthesizing proteins fused with GFP^64–66^. Our current study supports the conclusion that a CPP can broaden the range of applications of CPPs in Fungi.

In conclusion, we propose that the novel CPP Cupid might be a valuable candidate for further investigations concerning its ability to transport large protein cargo molecules into and throughout a great variety of cells across the kingdoms, which are free to refold into bioactive forms. Such capability would lend itself toward numerous applications within research and maybe future therapies.

## Supplementary information

Supplementary Information.

Supplementary Video 1.

Supplementary Video 2.

Supplementary Video 3.
