# Supplementary material for: Cupid, a cell permeable peptide derived from amoeba, capable of delivering GFP into a diverse range of species
Source: Sci Rep. 2020 Aug 13;10:13725. doi: 10.1038/s41598-020-70532-x (PMC7426420; doi:10.1038/s41598-020-70532-x)
Supplement: Supplementary file 1 — Supplementary Information. [file 41598_2020_70532_MOESM1_ESM.pdf]

Supplementary Figures

Manuscript title:

Cupid, a cell permeable peptide derived from amoeba, capable of delivering GFP into a diverse range of species.

Supplemental Figure 1.

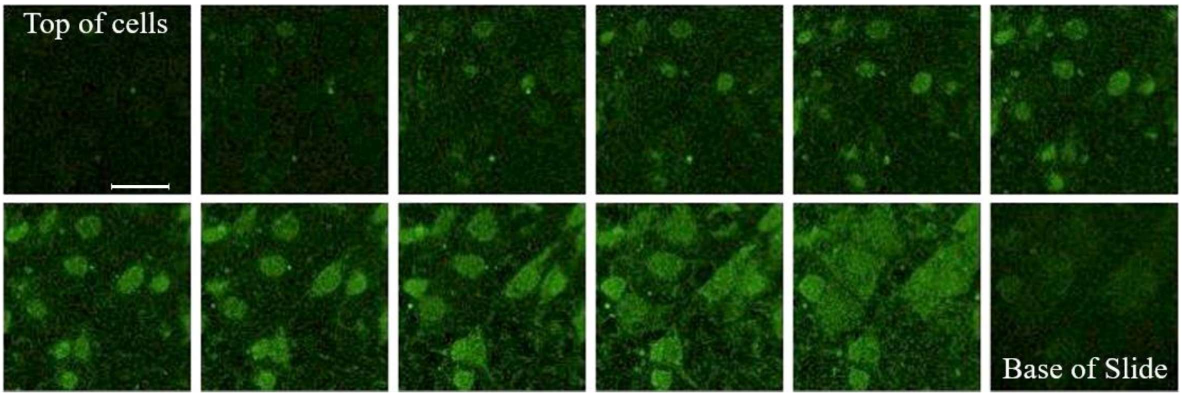

Supplemental Fig 1. Z-Slice green fluorescent confocal imaging of confluent mouse cardiomyocytes at 1 hour after 5 µM Cupid-GFP addition. Consecutive Z-Slices from top of cells are shown from left to right to the base of the slide. Scale bar 20µm

Supplemental Figure 2.

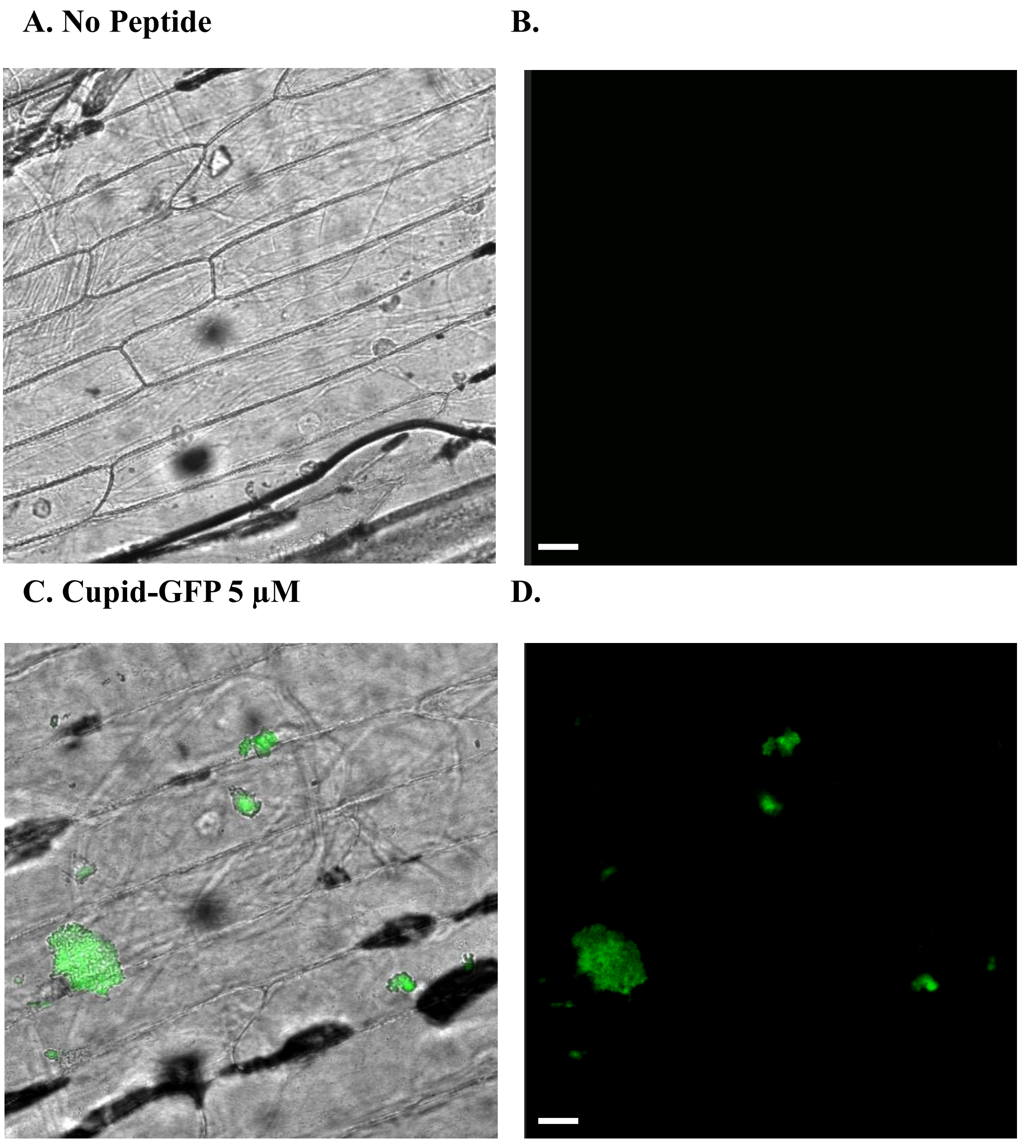

Supplemental Fig 2. Onion epidermis imaged 1 hour after addition of either water **(A, B)** or 5 µM native fluorescent Cupid-GFP **(C, D)**. Images **A** & **C** show merged phase contrast and green fluorescence channels. **B** & **D** show green fluorescence alone. Scale bar 50µm.

Supplemental Figure 3.

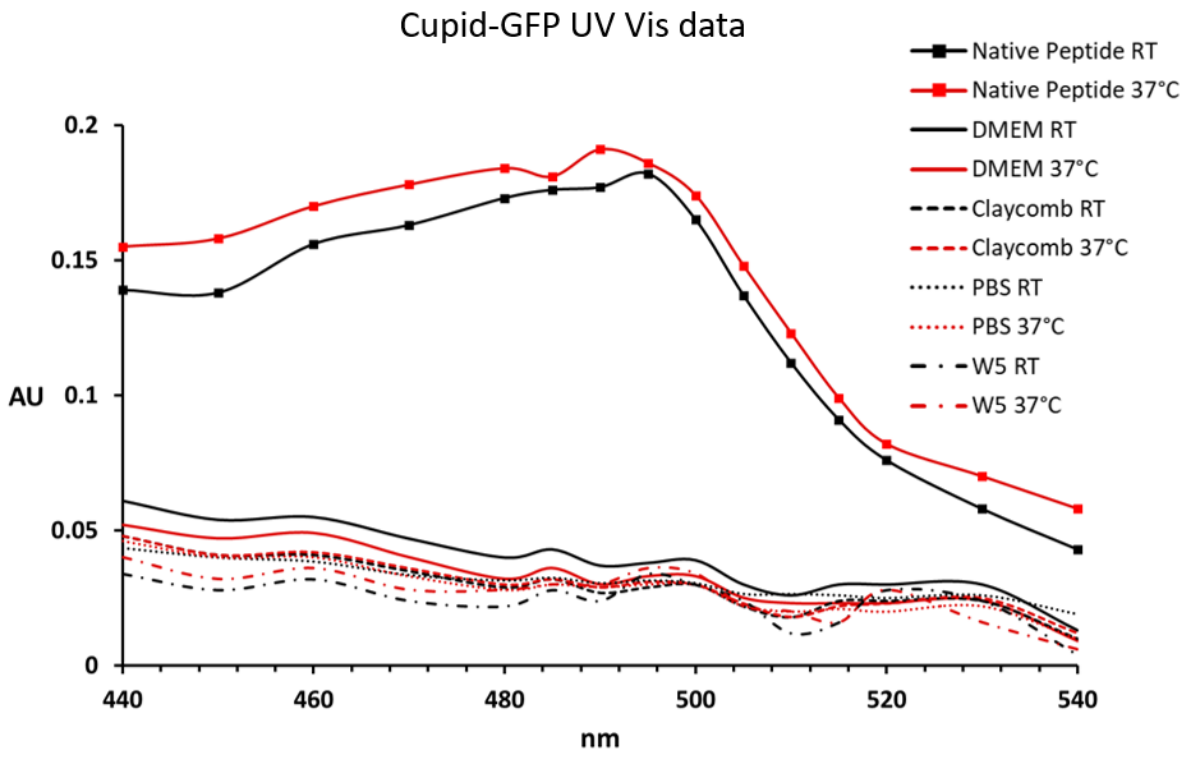

Supplemental Fig 3.
UV-Vis absorption spectra of denatured Cupid-GFP (50 µM) after incubation in a range of cell-culture media for over 24h at room temperature (RT) or 37°C. Native (folded and fluorescent) Cupid-GFP incubated in water at RT or 37°C for 24h also included. Y-axis: absorbance units, X-axis: nanometers.

Supplemental Fig 4.

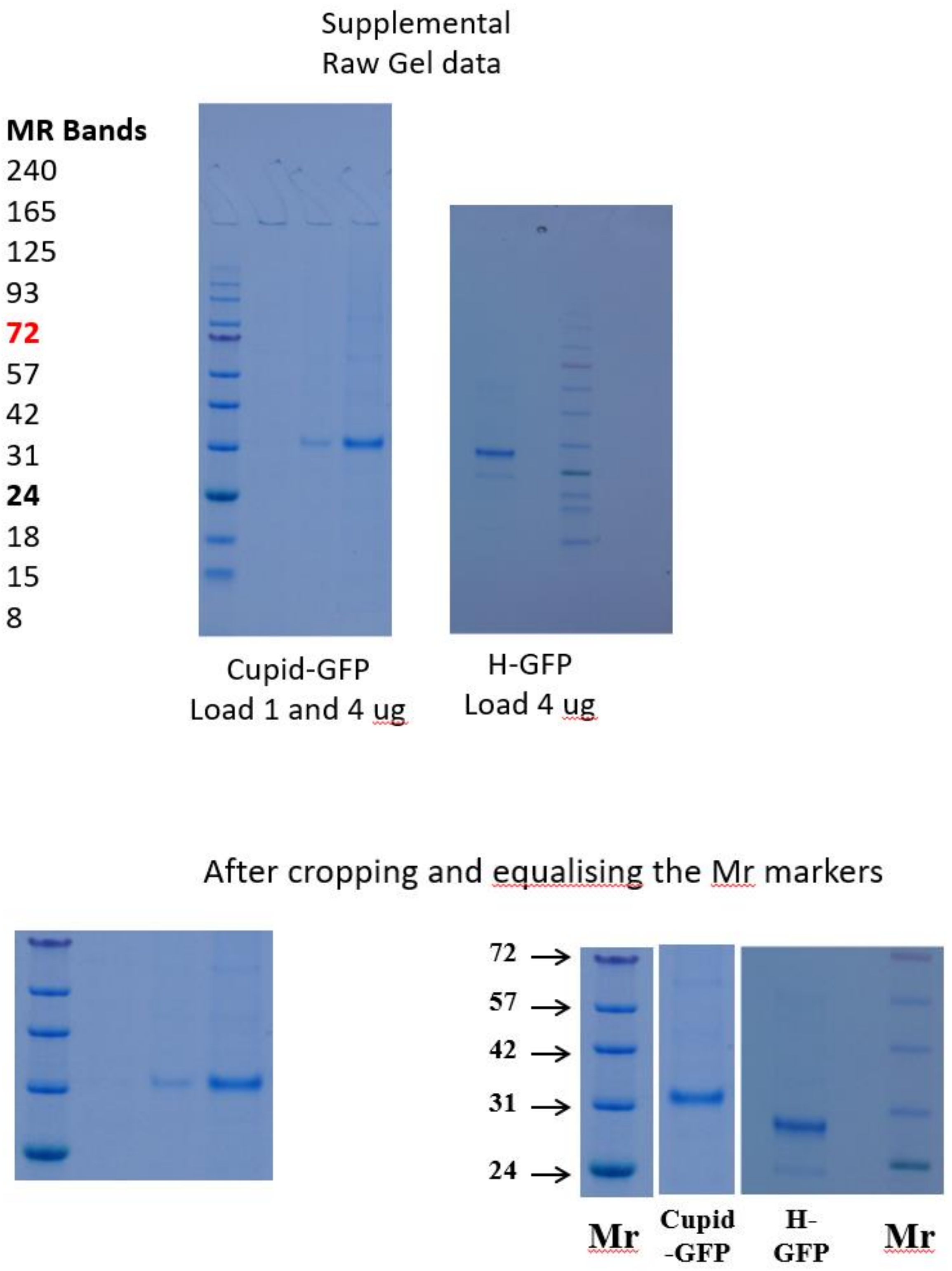

Supplemental Fig 4.
Full-length SDS-PAGE gels showing that purified Cupid-GFP and H-GFP migrated as single bands at the expected mass of of 32.3 kD and 29.2 kD respectively.

Supplemental Video. After treatment of a living *Phymocyces blakesleeanus* mycelia network within a 5 mm deep agar block with 5 µM Cupid-GFP for 90 minutes, a Z-plane time-lapse video (8 sec/frame) was taken over the course of 1 minute 42 sec. **Video 1** with phase contrast; **Video 2** with green fluorescent filters; and **Video 3** combined overlay.
